# The Model of Goal-Directed Behavior in Sports Participation: A Meta-Analysis Comparing Pre- and Post-COVID-19 Eras in the Republic of Korea

**DOI:** 10.3390/bs14070556

**Published:** 2024-07-01

**Authors:** Jung-Sup Bae, Weisheng Chiu, Sang-Back Nam

**Affiliations:** 1 Division of Academic Affairs, Konkuk University–Glocal Campus, Chungju-si 27478, Republic of Korea; bluelion10@kku.ac.kr; 2 Lee Shau Kee School of Business and Administration, Hong Kong Metropolitan University, Kowloon, Hong Kong, China; 3 Division of Sport Science, Hanyang University-ERICA Campus, Ansan 15588, Republic of Korea

**Keywords:** sports participation, physical activity, model of goal-directed behavior, meta-analysis, COVID-19 pandemic

## Abstract

The COVID-19 pandemic has had a significant impact on sports participation worldwide, including in Republic of Korea. Due to the restrictions and safety measures implemented to prevent the spread of the virus, many individuals have had to alter their sports participation. Therefore, this study conducted a meta-analysis based on studies applying the Model of Goal-directed Behavior (MGB) in sports participation to compare the pre- and post-COVID-19 eras in Republic of Korea. By analyzing 18 selected studies involving 5772 Korean respondents, the study investigates the relationships between MGB variables and sports consumption behaviors pre- and post-COVID-19. This study found that all correlations with the MGB were significant, indicating the applicability of the MGB in understanding sports participation in Republic of Korea. Moreover, the moderating effect of COVID-19 was identified in the relationships between subjective norms and desire and between perceived behavioral control and desire. The findings of this study contribute to a deeper understanding of the factors influencing sports participation in Republic of Korea. Additionally, the study provides valuable insights for sports managers and policymakers in Republic of Korea to develop strategies and interventions that can promote and support sports participation during challenging times.

## 1. Introduction

Understanding sports participation behavior is essential for marketers and businesses involved in the sports industry. It allows them to tailor their strategies and offerings to meet the needs and preferences of individuals in different contexts and circumstances [1,2,3]. Specifically, sports participation behavior involves individuals engaging in physical activities such as playing sports, participating in fitness training, or being involved in recreational pursuits like hiking or swimming. This type of behavior is characterized by the active involvement of individuals in sports-related activities, including organized sports, fitness training, and recreational pursuits [1]. Individuals’ engagement with various sporting disciplines has been recognized to have numerous benefits for physical health, mental well-being, and the development of social interactions and skills [4,5,6].

Especially in Republic of Korea, sports participation has been regarded as an important cultural and social activity [7,8]. Sport plays a significant role in the lives of people in Republic of Korea, with a strong emphasis on active participation [9]. In the context of Republic of Korea, sports participation behavior encompasses a wide range of activities, including organized sports, fitness training, and recreational pursuits [10,11,12]. This active involvement of individuals in sports-related activities has been linked to the promotion of physical and mental well-being, reflecting the cultural and social significance of sports in the country. Furthermore, the government of Republic of Korea has implemented various policies and initiatives to promote and support sports participation among its citizens, recognizing the positive impact of sports on individual health and community. The participation rate in sports and physical activities among the population had a significant increase from 54.8% to 66.6% between 2014 and 2019 [13]. Sports participation behavior is deeply ingrained in the cultural fabric of the nation and contributes to the promotion of a healthy and active lifestyle among Republic of Koreans [14,15].

However, the COVID-19 pandemic has had a significant impact on sports participation behavior in Republic of Korea [16]. Like other parts of the world, the outbreak led to widespread closures of sports facilities, cancellation of events, and restrictions on gatherings, significantly impacting participation in sports and physical activities in Republic of Korea [17]. As a result, understanding the shift in sports participation behavior pre- and post-COVID-19 has become crucial for stakeholders in the sports industry. The need to delve deeper into the evolving dynamics of sports participation behavior in the face of a global pandemic has necessitated research to identify the changes and challenges faced by individuals and organizations involved in sports consumption.

Previous studies have applied the model of goal-directed behavior (Perugini & Bagozzi, 2001, MGB) to understand sports participation behavior [18,19]. The MGB is a social psychological model that expands the theory of planned behavior by incorporating habitual, motivational, and affective factors into the decision-making process [20,21]. Researchers in the field of sports management have particularly highlighted the MGB as a valuable framework for examining sports participation behavior [18]. In contrast to other social psychology theories (e.g., theory of reasoned action and theory of planned behavior), the MGB takes into account the complexity and multidimensionality of individuals’ attitudes, social factors, beliefs, motivations, emotions, and habitual tendencies that shape their individuals’ intentions and behaviors [21,22,23]. As a result, research has found the MGB to be a useful tool for understanding and predicting individuals’ participation in sports and physical activities.

Nevertheless, it is evident that the pandemic has not only changed the way individuals engage with sports but has also brought about a shift in their motivations and decision-making processes. Factors such as health and safety concerns and the influence of social and peer interactions on sports consumption have become paramount in the post-COVID-19 era [24,25]. Moreover, the psychological and emotional aspects of sports participation behavior have come to the forefront, with individuals seeking a sense of connection, community, and escapism through their sports-related activities. Understanding these underlying motivations and the interplay of various socio-environmental factors is crucial for comprehensively comprehending the shifting landscape of participation in sports and physical activities.

The COVID-19 pandemic has undoubtedly altered the landscape of sports and physical activities. In order to fully capture the impact of COVID-19 on sports consumer behavior, it is crucial to conduct a thorough analysis comparing the pre-COVID-19 era with the post-COVID-19 era. Accordingly, this study aimed to examine sports participation behavior in the pre- and post-COVID-19 eras, using the model of goal-directed behavior as a framework. By conducting a meta-analysis, the researchers aimed to provide a comprehensive and evidence-based understanding of the changes in sports participation behavior during the pandemic within the Republic of Korean context. The findings of this meta-analysis will not only provide valuable insights for marketers and businesses in the sports industry but will also contribute to the academic understanding of consumer behavior in the context of sports, particularly in the challenging and transformative times brought about by the COVID-19 pandemic.

## 2. Literature Review

### 2.1. Model of Goal-Directed Behavior

**Figure 1:**
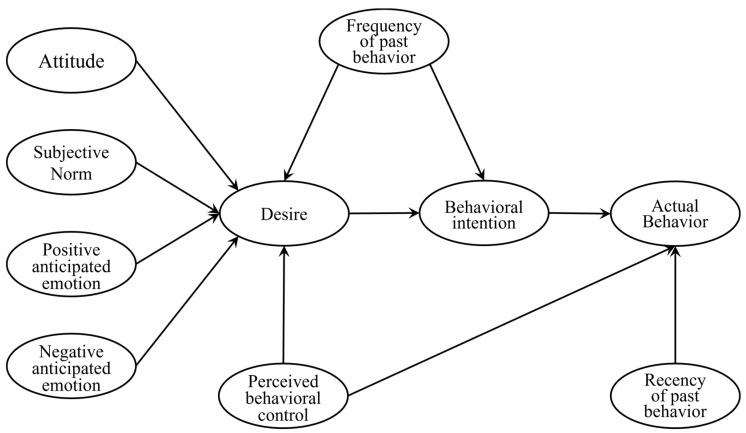
Model of goal-directed behavior [20].

The model of goal-directed behavior (MGB) is a theoretical framework that incorporates various psychological and social factors to explain and predict human behavior. The MGB is an extension of the theory of planned behavior (TPB, [26]), incorporating additional elements to provide a more comprehensive understanding of goal-directed behaviors. Specifically, the TPB suggests that a decision and intention are determined by three key factors: attitudes, subjective norms, and perceived behavioral control [26]. The MGB expands on the TPB by including ‘anticipated emotions’ as a psychosocial predictor and inserting ‘desire’ between the psychosocial predictors and intentions [27]. This extension allows for a more nuanced exploration of the emotional and motivational aspects that influence goal-directed behaviors [28]. Moreover, the MGB incorporates desire and emotional components, such as positive emotions, into the previous TPB, highlighting the linkages among human emotions, affection, past behaviors, and goal-directed behaviors. Because of the inclusion of these additional elements, the MGB offers a more comprehensive understanding of consumer behavior and the factors that influence their decisions and actions [27,28]. The MGB is shown in Figure 1.

### 2.2. MGB in Sports Participation Behavior

The MGB has been widely applied across different disciplines to study various aspects of consumer decision-making and behavior. In particular, the MGB has been applied to explore sports participation, providing valuable insights into the factors influencing individuals’ intentions and behaviors in sports-related activities. For example, Esposito, et al. [18] utilized the MGB to examine the factors influencing individuals’ intentions to engage in physical activity. Moreover, Won, Kim, and Bae [19] applied the MGB to explore individuals’ desire and intention to participate in an inner-city ‘running crew’ among social runners. These studies demonstrate the applicability and effectiveness of the MGB in understanding and predicting sports participation behavior.

However, it is important to note that there is a research gap when it comes to the inconsistent relationship within the MGB in the context of sports participation behavior. For example, the relationship between negative anticipated emotion and desire showed mixed results across different studies [18,19]. While previous studies have successfully utilized the MGB to understand and predict sports participation behavior, there is a lack of comprehensive research on the inconsistencies within the model when applied to the dynamics of sports participation behavior.

### 2.3. COVID-19 and Sports Participation Behavior

The outbreak of the COVID-19 pandemic has had a significant impact on sports participation behavior [24,25]. The evolving socio-environmental factors, such as health and safety concerns, government-imposed restrictions, and the influence of social and peer interactions on sports participation, have likely introduced inconsistencies within the traditional application of the MGB. These inconsistencies may manifest in the form of shifts in anticipated emotions, desires, and habitual tendencies among individuals in response to the changing landscape of sports participation during the pandemic.

Therefore, further research is needed to explore the specific inconsistencies within the application of the MGB in the context of sports participation behavior, particularly in the post-COVID-19 era. Understanding these inconsistencies will not only contribute to the theoretical refinement of the model but will also provide practical implications for businesses and marketers in the sports industry. By addressing this research gap, the findings can offer insights to enhance the effectiveness of marketing strategies and offerings in response to the dynamic changes in sports participation behavior during and after the COVID-19 pandemic.

## 3. Methods

### 3.1. Search Strategy and Selection of Studies

**Figure 2:**
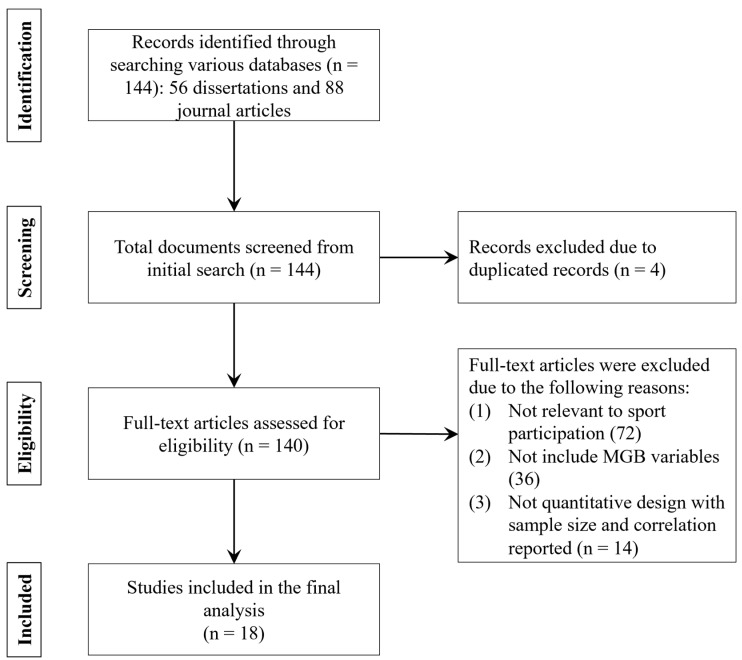
Flow chart for the meta-analysis.

The search strategy for relevant studies was conducted following the guidelines of Preferred Reporting Items for Systematic Reviews and Meta-Analyses (PRISMA), which includes identification, screening, eligibility assessment, and study inclusion [29]. First, we searched for relevant studies published in Korean and English using various databases, including the National Assembly Digital Library (http://dl.nanet.go.kr), academic research information service (http://www.riss.kr), and Korean academic information (http://kiss.kstudy.com). As this study targeted the research context in Republic of Korea, these major academic databases in Republic of Korea were chosen. Moreover, these databases also include studies published in English and Korean. Therefore, it allows researchers to identify both English and Korean studies conducted in the context of Republic of Korea. More specifically, following Chiu and Cho’s [30] strategy, we searched relevant literature by using the combination of the following keywords: “model of goal-directed behavior” AND “sport”, “model of goal-directed behavior” AND “physical activities”, as well as “model of goal-directed behavior” AND “sports participation”. As a result, a total of 144 articles (56 articles from dissertations and 88 articles from journals) were retrieved from the initial search. Moreover, throughout the screening process, four studies were excluded due to the duplicated records. Next, 140 studies were evaluated during the eligibility process according to the following criteria: (1) studies must be relevant to sports participation. (2) Studies must use MGB as the theoretical framework and include all/partial MGB measures; (3) studies must have a quantitative design that includes both sample size and correlation. In addition, estimation of effect size requires both sample size and correlation; therefore, only quantitative studies with both sample size and correlation coefficients were considered for inclusion in the meta-analysis. As a result, 14 studies were excluded due to the missing information on sample size or correlation. Consequently, 18 studies were included, covering 5772 Korean respondents. Figure 2 depicts a flow chart outlining the study selection process.

### 3.2. Coding Procedure

Coding was performed by the main author and a doctoral degree holder with experience in meta-analysis. Following the guidelines of Brown, et al. [31], the article information was coded for all selected articles (*n* = 18), including author, year of publication, and sample size, in the Comprehensive Meta-Analysis (CMA) Version 3. software. Moreover, the correlation coefficients between all relationships between the MGB variables were coded. In addition, the types of sports activities and pre- or post-COVID-19 were coded. Notably, although some studies were published after the outbreak of the COVID-19 pandemic, the data were collected prior to the pandemic. Therefore, these studies were coded as pre-COVID-19 [32,33]. In particular, the effect sizes of each pairwise relationship in the meta-analysis were calculated using the correlation coefficients between the MGB variables and the sample size of each study. It is important to mention that the studies reviewed in this meta-analysis did not involve observing actual behavior. In addition, the recency of past behavior and frequency of past behavior in the MGB were not taken into account due to the small sample sizes. This means that the relationships related to these three variables (i.e., actual behavior, frequency of past behavior, and recency of past behavior) could not be analyzed.

### 3.3. Analytic Procedure

Data analysis was performed using the Comprehensive Meta-Analysis version 3 software. Following Hedges and Olkin’s [34] analytical approach, the effect size of each paired relationship was determined by converting the correlation coefficient *r* into Fisher’s z-scores. A random-effect model was utilized to synthesize the overall effect sizes from various studies conducted independently in different settings. Furthermore, both the point estimates of effect sizes and their corresponding 95% confidence intervals for estimated correlations were reported. Moreover, the correlation effect magnitudes were interpreted based on Cohen’s guidelines, which classify effect sizes into three categories: small for values ranging from 0.10 to 0.30, medium for values ranging from 0.30 to 0.50, and large for values between 0.50 and 1.00 [35]. In addition, publication bias occurs when published studies do not sufficiently represent the entire study. Therefore, in order to assess publication bias, we used funnel plots, Egger’s test, and Rosenthal’s fail-safe N [36,37].

In the following stage, the mixed-effects model was employed to conduct the moderator analysis. This model utilized a random effects model within each subgroup and a fixed effects model across subgroups [36]. Homogeneity estimates (*Q_B_* test) were computed. The *Q_B_* test is a method used to assess the relative homogeneity of an effect by calculating the *Q_B_* statistic, which follows a chi-square distribution with *k* − 1 degrees of freedom. The obtained *Q_B_* statistic showed statistical significance at *p* < 0.001, indicating heterogeneity in the effect. Specifically, COVID-19 was used as the moderator to examine whether the effect sizes differed between the pre- and post-COVID-19 eras.

## 4. Results

### 4.1. Characteristics of Included Studies

A total of 18 studies consisted of 16 journal articles and 2 dissertations published between 2013 and 2023. One study was published in English language and the rest (*n* = 17) were published in Korean. Overall, each study’s sample size ranged from 238 to 446 (M = 320.67, SD = 64.22), and the total sample size of included studies was 5772. Moreover, there were 8 studies published in the pre-COVID-19 era and 10 studies published in the post-COVID-19 era. In addition, sports activities include fitness (*n* = 5), golf (*n* = 3), running (*n* = 1), tennis (*n* = 1), scuba diving (*n* = 1), horseback riding (*n* = 1), VR sports activities (*n* = 1), indoor adventure activities (*n* = 1), and outdoor activities (*n* = 1). However, three studies did not specify the type of sports activities (see Appendix A).

### 4.2. Meta-Analysis

The results of the meta-analysis of the six pairwise correlations with the MGB are reported in Table 1. A random effects model was examined for the effect sizes of all the correlations. Table 1 showed that all pairwise relationships were significant, ranging from 0.361 to 0.671 (*p* < 0.001). Specifically, the DSE–INT correlation showed the most substantial effect size (0.671), followed by PAE–DES (0.619) and ATT–DES (0.525), both indicating strong effect sizes [35]. Moreover, the correlations of SN–DES (0.486), PBC–DES (0.435), and NAE–DES (0.361) showed moderate effect sizes [35].

**Figure 3:**
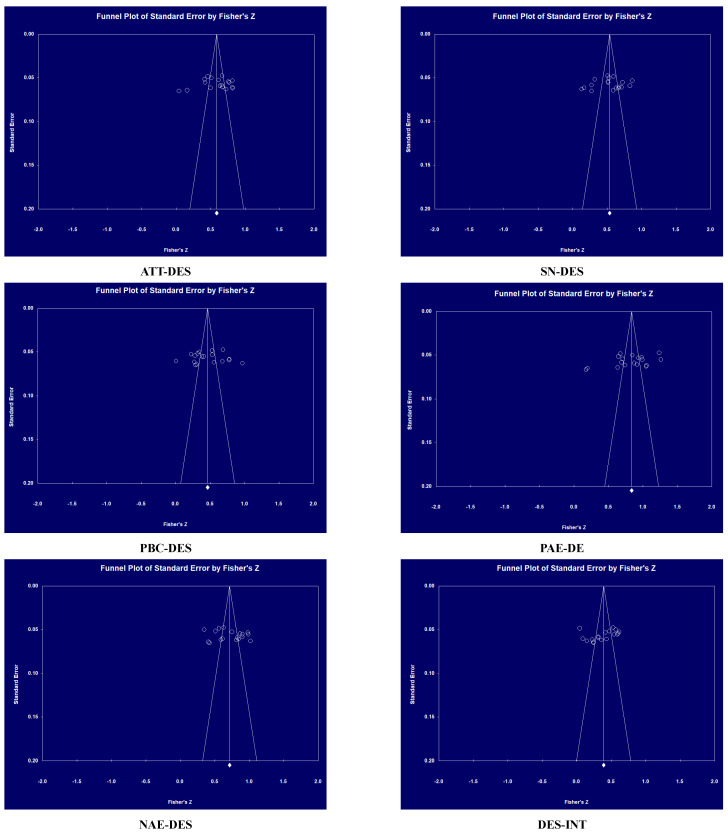
Funnel chart.

In addition, publication bias was examined by funnel plots and Rosenthal’s fail-safe N. First, the preliminary judgment about whether publication bias existed was examined using a funnel plot. A better symmetry effect indicates lower bias, with a symmetrical distribution of inverted funnel effect sizes obtained from a good set of literature for meta-analysis. The funnel plot in Figure 3 shows six pairwise relationships of effect sizes concentrated at the top with a symmetrical distribution pattern around the mean value, indicating that no publication bias exists in this study. In order to test publication bias more accurately, Rosenthal’s fail-safe N was adopted [36,37]. As a result, the fail-safe *N* for each significant effect size is greater than the recommended threshold, indicating that the overall findings are robust and unlikely to be significantly influenced by unpublished studies.

### 4.3. Moderator Analysis

Because the values of *Q_W_* were significant for all pairwise relationships, it indicated the substantial heterogeneity between the effect sizes of different studies. This finding suggests the existence of potential moderating variables in different studies. Therefore, we conducted a moderator analysis to examine the impact of COVID-19 on the pairwise relationships within the MGB. Specifically, the studies were split into two groups: pre-COVID-19 era (8) and post-COVID-19 era (10), and the effect sizes were compared between the two groups. The results of the moderator analysis are reported in Table 2. As shown in Table 2, significant differences were found in the effect sizes of two pairwise relationships (i.e., SN–DSE and PBC–DSE) between the pre- and post-COVID-19 eras. Specifically, the correlation of SN–DSE was stronger in the pre-COVID-19 era, while the correlation of PBC–DSE was stronger in the post-COVID-19 era.

## 5. Discussion

To reconcile the results of relevant studies and clarify the relationships between the MGB variables, a meta-analysis was conducted to synthesize the empirical results of studies applying the MGB to explore sports participation in Republic of Korea. This study provides statistical evidence supporting the application of the MGB in understanding sports participation in Republic of Korea. Moreover, this study identified the influential role of COVID-19 on the relationships within the MGB. The findings of this study advance our understanding of the MGB and its applicability in the specific context of sports participation in Republic of Korea. Specifically, 18 studies (N = 5772) were included in the meta-analysis, and the results indicate that there is no publication bias present in the study. The results of the meta-analysis found significant effect sizes between the MGB variables. Moreover, the moderator analysis revealed that the impact of COVID-19 differed across pairwise relationships of SN–DSE and PBC–DSE within the MGB. The results summary, theoretical and practical implications, limitations, and future research directions are discussed below.

### 5.1. Results Summary

The meta-analysis revealed that all relationships with the MGB were significant, with medium to strong effect sizes. Specifically, the relationship between desire and behavioral intention was found to be strongest, consistent with the results of previous meta-analyses on the MGB in different contexts, such as tourism and hospitality, as well as consumer behavior in marketing [30,38]. Moreover, the relationships between desire and its antecedents (i.e., attitude, subjective norm, perceived behavioral control, positive and negative anticipated emotions) revealed similar patterns with previous studies. In particular, correlations between volitional factors (i.e., attitude and subjective norms) and desires were found to be moderately strong. Meanwhile, nonvolitional factors (i.e., perceived behavioral control) and desire had a moderate effect size [30,38]. Regarding emotional factors, positive anticipated emotions showed a stronger relationship with desire compared to negative anticipated emotions [30,38]. The findings suggest that the MGB is applicable to understanding sports participation in Republic of Korea, and the relationships within the model are significant with medium to strong effect sizes.

Moreover, a series of moderator analyses revealed that the relationships within the MGB were influenced by the COVID-19 pandemic. That is, the magnitudes of some MGB relationships vary significantly between the pre- and post-COVID-19 eras in Republic of Korea. Specifically, the pairwise relationships of SN–DSE and PBC–DSE were moderated by the COVID-19 pandemic. The effect size of the SN–DSE relationship was stronger in the pre-COVID-19 era compared to the post-COVID-19 era, suggesting that subjective norms had a greater influence on the desire for sports participation before the pandemic [39]. That is, individuals were more likely to be influenced by the social expectations and norms surrounding sports participation prior to the COVID-19 outbreak. On the other hand, the effect size of the PBC-DSE relationship was stronger in the post-COVID-19 era, indicating that perceived behavioral control had a greater impact on the desire for sports participation during the pandemic. In other words, individuals’ belief in their ability to engage in sports activities despite the challenges posed by the pandemic became a more influential factor in driving their desire for sports participation [40,41]. These findings highlight the dynamic nature of sports participation behaviors in response to external factors such as the COVID-19 pandemic. The pandemic has evidently reshaped individuals’ desires related to sports participation, emphasizing the need for a nuanced understanding of psychological and environmental influences on behavior.

### 5.2. Theoretical Implications

The findings of this study provide valuable insights into the theoretical implications of understanding sports participation behavior, particularly in the context of the COVID-19 pandemic. The research highlights the dynamic nature of individuals’ desires related to sports participation and emphasizes the need for a nuanced understanding of psychological and environmental influences on behavior.

One key theoretical implication is the importance of perceived behavioral control in driving the desire for sports participation during the pandemic. The findings suggest that individuals’ belief in their ability to engage in sports activities despite the challenges posed by the pandemic became a more influential factor in shaping their desire for sports participation [42,43]. This underscores the significance of self-efficacy and perceived control in influencing behavior, especially in times of crisis. Moreover, the study points to the evolving role of social expectations and pressures (i.e., subjective norms) surrounding sports participation [44,45]. Prior to the COVID-19 outbreak, individuals were more likely to be influenced by these social factors. However, in the post-COVID-19 era, the effect size of the PBC-DSE relationship was stronger, indicating a shift toward greater emphasis on perceived behavioral control in driving sports participation desires [46]. This highlights the adaptive nature of individuals’ behavior in response to external factors such as a global pandemic [12,47].

Furthermore, the research underscores the need for further exploration of the specific inconsistencies within the application of the MGB in the context of sports participation behavior, particularly in the post-COVID-19 era [38]. By addressing these research gaps, the findings can contribute to the theoretical refinement of the model and provide practical implications for businesses and marketers in the sports industry. Understanding these inconsistencies can enhance the effectiveness of marketing strategies and offerings in response to the dynamic changes in sports participation behavior during and after the COVID-19 pandemic. In conclusion, this meta-analysis provides valuable insights into the theoretical implications of understanding sports participation behavior, particularly in the context of Republic of Korea, during both the pre- and post-COVID-19 eras.

### 5.3. Practical Implications

The findings of this study offer practical implications for businesses and marketers in the sports industry, particularly in the post-COVID-19 pandemic. Understanding the evolving dynamics of sports participation behavior can guide businesses in developing targeted marketing strategies and offerings to meet the changing needs and desires of consumers. By addressing the research gaps and inconsistencies within the application of the MGB, businesses can refine their approaches to engage effectively with consumers and adapt to the shifting landscape of sports participation behavior. First, organizations and marketers should acknowledge the increased emphasis on perceived behavioral control in driving sports participation desires, particularly in the post-COVID-19 era. They should focus on empowering individuals and providing them with the necessary resources and support to overcome barriers and engage in sports activities [15,46]. For example, organizations can offer flexible membership options, provide easy access to sports facilities, and implement safety protocols to instill confidence in individuals.

Second, this study underscores the significance of adapting to external factors, such as the COVID-19 pandemic, to meet consumer needs in the sports industry. Although the COVID-19 pandemic has come to an end, other public health crises or unforeseen circumstances may occur in the future. Therefore, organizations and marketers should be prepared to adapt their strategies and offerings to cater to changing circumstances. They should embrace digital platforms and social media to reach a wider audience and engage with consumers remotely, as these channels have proven to be effective in sustaining interest and motivating individuals to engage in sports activities, even during challenging times.

### 5.4. Limitations and Future Research

Despite the importance of this study’s findings, there are some limitations that should be acknowledged. First, two factors within the MGB, frequency of past behavior and actual behavior, were not included in the meta-analysis due to the small number of studies available. Many studies did not report the correlation coefficients of the frequency of past behavior and actual behavior with other MGB variables. Second, the study focused on Republic of Korea as a specific cultural and geographical context. Therefore, the generalizability of the findings to other countries and cultures may be limited. Future research should consider conducting cross-cultural studies to explore potential variations in sports participation behavior and consumer preferences in different cultural contexts. Furthermore, while the authors attempted to include all the relevant studies, there is always a possibility of publication bias, as studies with non-significant results are less likely to be published. Fortunately, the results of publication bias tests were not significant, indicating that the meta-analysis results are robust and not skewed by publication bias [37].

## 6. Conclusions

The meta-analysis on the MGB in sports participation in Republic of Korea highlights the significance of understanding and adapting to changing circumstances, such as the COVID-19 pandemic, to meet consumer needs in the sports industry. The meta-analysis found significant correlations within the MGB variables, indicating the effectiveness and applicability of the MGB in understanding sports participation behaviors in the context of Republic of Korea. The study emphasizes the importance of adapting marketing strategies to empower individuals and meet their changing needs, particularly in the post-COVID-19 era. By addressing research gaps and inconsistencies, businesses can refine their approaches to engage effectively with consumers and navigate the evolving landscape of sports participation behavior. Through a systematic and comprehensive literature search, 14 studies were included for meta-analysis. These findings provide valuable insights for sports managers and policymakers to develop targeted interventions that promote and support sports participation in Republic of Korea.

## Figures and Tables

**Table 1:** Meta-analysis results.

Pairwise Relationship	*k*	*n*	Effect Size (*r*)	95% CI	Z Value	*p* Value	*Q_W_*	*I* ^2^	Fail-Safe *N*
ATT–DES	18	5772	0.525	[0.453, 0.590]	12.097	0.000	225.128 ***	92.449	9105
SN–DES	18	5772	0.486	[0.409, 0.556]	10.837	0.000	227.840 ***	92.539	7399
PBC–DES	18	5772	0.435	[0.344, 0.518]	8.514	0.000	290.034 ***	94.139	5733
PAE–DES	18	5772	0.619	[0.554, 0.676]	14.366	0.000	245.380 ***	93.072	3785
NAE–DES	18	5772	0.361	[0.284, 0.434]	8.536	0.000	189.033 ***	91.007	3909
DES–INT	18	5772	0.671	[0.593, 0.736]	12.323	0.000	416.843 ***	95.922	7820

Note: *k* = number of studies; *n* = cumulative sample size; *r* = the magnitude of estimated correlations; CI = confident interval; ATT = attitude; DES = desire; SN = subjective norm; PBC = perceived behavioral control; PAE = positive anticipated emotions; NAE = negative anticipated emotions; INT = behavioral intention; *Q_w_* is an intragroup heterogeneity test statistic, *** p < 0.001.

**Table 2:** Results of Moderator analysis.

Pairwise Relationship	Moderator: COVID-19	*k*	*N*	Effect Size (*r*)	95% CI	Z Value	*p* Value	*Q_W_*	*Q_B_*
ATT–DES	Pre- COVID-19	8	2574	0.516	[0.414, 0.605]	8.571	0	78.795	0.055
	Post- COVID-19	10	3198	0.533	[0.424, 0.626]	8.265	0	145.961	
SN–DES	Pre- COVID-19	8	2574	0.555	[0.498, 0.609]	15.344	0	28.355	3.928 *
	Post- COVID-19	10	3198	0.424	[0.293, 0.540]	5.871	0	168.454	
PBC–DES	Pre- COVID-19	8	2574	0.331	[0.199, 0.451]	4.753	0	93.135	4.931 *
	Post- COVID-19	10	3198	0.511	[0.407, 0.602]	8.359	0	128.587	
PAE–DES	Pre- COVID-19	8	2574	0.614	[0.500, 0.707]	8.457	0	127.289	0.017
	Post- COVID-19	10	3198	0.623	[0.539, 0.694]	11.288	0	118.079	
NAE–DES	Pre- COVID-19	8	2574	0.421	[0.305, 0.525]	6.583	0	82.663	2.165
	Post- COVID-19	10	3198	0.311	[0.215, 0.402]	6.114	0	78.383	
DES–INT	Pre- COVID-19	8	2574	0.670	[0.542, 0.767]	7.789	0	188.335	0.001
	Post- COVID-19	10	3198	0.672	[0.563, 0.757]	9.057	0	228.301	

*Q_B_* is an intergroup heterogeneity test statistic, * p < 0.05.

## Data Availability

The data presented in this study are available on request from the corresponding author.

## Appendix A

**Table A1:** Studies included in meta-analysis.

No.	Authors	Sample Size	Type of Sport Activities	Pre- or Post-COVID-19
1	Dong, et al. [48]	277	Golf	Pre-COVID-19
2	Lim and Lee [49]	330	Scuba diving	Pre-COVID-19
3	Lee, et al. [50]	327	Horseback riding	Pre-COVID-19
4	Nam [51]	340	Fitness activities	Pre-COVID-19
5	Lee, et al. [52]	288	Fitness activities	Pre-COVID-19
6	Lee [53]	402	VR sport activities	Pre-COVID-19
7	Chang, Lee, and Moon [32]	365	Indoor adventure activities	Pre-COVID-19
8	Won, Kim, and Bae [33]	254	Fitness activities	Pre-COVID-19
9	Park [54]	272	N/A	Post-COVID-19
10	Lee, et al. [55]	263	Golf	Post-COVID-19
11	Lee, et al. [56]	446	Fitness activities	Post-COVID-19
12	Jung, et al. [57]	377	Tennis	Post-COVID-19
13	Park, et al. [58]	238	Golf	Post-COVID-19
14	Bae and Kim [59]	296	Fitness activities	Post-COVID-19
15	Jung [60]	356	N/A	Post-COVID-19
16	Jung and Kim [61]	429	N/A	Post-COVID-19
17	Kim, et al. [62]	267	Outdoor activities	Post-COVID-19
18	Won, Kim, and Bae [19]	245	Running	Post-COVID-19
